# Cultural Similarities and Differences in Social Discounting: The Mediating Role of Harmony-Seeking

**DOI:** 10.3389/fpsyg.2018.01426

**Published:** 2018-08-08

**Authors:** Keiko Ishii, Charis Eisen

**Affiliations:** ^1^Department of Psychology, Graduate School of Informatics, Nagoya University, Nagoya, Japan; ^2^Department of Psychology, Graduate School of Humanities, Kobe University, Kobe, Japan

**Keywords:** social discounting, culture, harmony-seeking, hyperbolic with exponent model, gains and losses

## Abstract

One’s generosity to others declines as a function of social distance, which is known as social discounting. We examined cultural similarities and differences in social discounting and the mediating roles of the two aspects of interdependence (self-expression and distinctiveness of the self) as well as the two aspects of independence (harmony-seeking and rejection avoidance). Using the same procedure that previous researchers used to test North Americans, Study 1 showed that compared to North Americans, Japanese discount more steeply a partner’s outcomes compared to their own future outcomes, whereas the decrease in the subjective value of the partner’s outcomes accelerates less as a function of social distance. To examine the cultural similarities and differences in social discounting in more detail, Study 2 tested Japanese and Germans and found that the hyperbolic with exponent model fitted the participants’ discounting behaviors better than the other models, except for the loss condition in Germans where the utility of the *q*-exponential model was indicated. Moreover, although the social discounting rate was higher in Japanese than in Germans, the cultural difference was limited to the gain frame. However, the decline in a person’s generosity accelerated less as a function of social distance in Japanese than in Germans. Furthermore, the cultural difference in the social discounting in gains was mediated by the level of harmony-seeking, which was higher in Germans than in Japanese. Implications for individuals’ generosity against the backdrop of cultural characteristics are discussed.

## Introduction

Humans are unique in their formation of cooperative relationships with unrelated individuals and living within a group. Specifically, other-regarding motives are crucial, as they promote cooperative relationships and altruistic behaviors. However, the motives are likely influenced by social distance, and therefore people are not always generous to everyone. Whereas people generally behave generously to close others, the tendency to generosity decreases for distant others (Hoffman et al., 1996; Jones and Rachlin, 2006; Rachlin and Jones, 2008; Goeree et al., 2010). Moreover, the variation in the social orientation of independence and interdependence across cultures also influences other-regarding motives (Yamagishi, 1988; Buchan et al., 2006; Strombach et al., 2014). In the present research, we examined the influence of social distance and cultures on other-regarding motives in the social discounting framework by comparing mathematical models. We also tested a hypothesis that generosity toward others increases as a function of harmony-seeking orientation, which differs across cultures.

### Social Discounting

People generally tend to discount future outcomes in exchange for immediate but smaller gains. Previous research examined the tendency of delay discounting by asking people to choose to either receive a stable amount of money with a specified delay or to receive a smaller amount of money immediately. Empirical evidence has suggested that the decline in the subjective value of future outcomes is likely steeper in the early delay phase and becomes more gradual as the delay gets longer (Ainslie, 1975; Mazur, 1987). Suppose that there are two alternatives. One is to receive $450 immediately, whereas the other is to receive $500 after 1 week. In this case, people will tend to prefer to receive $450 immediately. On the other hand, if people are asked to choose either to receive $450 after 5 years or to receive $500 after 5 years and 1 week, they will tend to prefer to receive $500 after 5 years and 1 week. People’s preferences for the two options are thus reversed in spite of the same length of delay (i.e., 1 week). Given such time-inconsistent choice behavior, it has been pointed out that a hyperbolic function better describes an individual’s delay discounting, compared to an exponential model assuming that the subjective value of future outcomes declines in a time-consistent manner (Kirby, 1997). Moreover, previous research (e.g., Laibson, 1997) has also proposed a quasi-hyperbolic discount model (sometimes called a quasi-exponential discount model) explaining such time-inconsistent choice behavior based on internal conflicts between “selves” having two or more exponential discount rates stocked within a single individual. Nevertheless, it has been suggested that models assuming a hyperbolic function (e.g., the *q*-exponential discount model and the hyperbolic with exponent model) fit individuals’ discounting behaviors better than the quasi-hyperbolic discount model (Takahashi, 2008; Ishii et al., 2018).

The social discounting framework focuses on choices between individuals who differ in social distance (i.e., a person and another person at a given social distance), instead of intertemporal choices. Jones and Rachlin (2006) assumed that social discounting would be related to delay discounting based on an expected positive association between other-regarding motives and self-control. Adopting the delay-discounting framework, Jones and Rachlin (2006) asked participants to choose that either another person receives a stable amount of money or they receive a smaller amount of money by manipulating the distances between the giver and the receiver. The scholars then compared an exponential discounting function and a hyperbolic discounting function to see which fits the behavioral data of social discounting better. The exponential discounting function and the hyperbolic discounting function are written, respectively, as follows:

(1) $$V(N)=\frac{V(0)}{\exp(kN)}$$

(2) $$V(N)=\frac{V(0)}{1+kN}$$

where *N* is the social distance, *V*(*N*) is the subjective value of a reward (or payment) when the receiver is a person at *N*, and *k* is a free parameter that represents the discount rate. The findings indicate that as in the case of delay discounting, the hyperbolic discount function is more suitable for explaining the tendency that an individual’s generosity to others is discounted by social distance. Concretely, the decline in the subjective value of the outcomes that another person receives is inconsistent across people who differ in social distance: The decline is likely steeper in early phase of social distance, whereas it becomes more gradual as social distance grows larger.

After Jones and Rachlin’s (2006) study, Rachlin and Jones (2008) adopted a more general form of the hyperbolic equation to explain the influence of social distance on an individual’s generosity to others. The equation is

(3) $$V(N)=\frac{V(0)}{1+kN^{S}}$$

where an exponent *s* is added to the social distance. *s* is a power-function parameter that suggests individual differences in the sensitivity of *V*(*N*)/*V*(0) to *N*. When *s* = 1, the equation is identical to the hyperbolic equation (2). However, when *s* is less than 1, the decrease in *V*(*N*)/*V*(0) over the course of the social distance diminishes faster than in the (simple) hyperbolic model. In Rachlin and Jones’s (2008) study (Experiment 1), *s* was 1.03, and the hyperbolic function was almost congruent with that reported in Jones and Rachlin (2006), which was based on the hyperbolic equation (2).

Takahashi (2010) proposed a *q*-exponential social discounting model based on Tsallis’ statistics. The equation is

(4) $$V(N)=\frac{V(0)}{\exp_{q}(kN)}=V(0)/{\left[1+(1-q)kN\right]}^{1/(1-q)}$$

where exp*_q_*(*x*), which is equal to ${\left[1+(1-q)x\right]}^{\frac{1}{1-q}}$, is a *q*-exponential function. When a parameter *q* = 1, Equation (4) expresses V (N) = V (0)^∗^ exp (-*kN*), which is equal to exponential model (1). When *q* = 0, Equation (4) expresses V (N) = V (0)/(1 + kN), which is equal to hyperbolic model (2). As a result, 1–*q* indicates the extent to which a person discounts another person’s reward (or payment) inconsistently depending on different social distances. This means that the decrease in the subjective value of another person’s reward (or payment) is more inconsistent as 1–*q* becomes larger (i.e., *q* becomes smaller). Takahashi (2013) examined social discounting in gain and loss and found that social loss is discounted less than social gain as the social distance increases. When he compared exponential, hyperbolic, and *q*-exponential models, he found that whereas the hyperbolic model fitted best to social gain, the *q*-exponential model fitted best to social loss. In the gain and loss frames, the *q*-values were smaller than 1, suggesting inconsistent social discounting across people differing in social distance.

### Cultural Differences in Social Discounting and Other-Regarding Motives

Previous researchers have suggested that culture influences interpersonal choices, which have not been fully investigated, however (Weber and Morris, 2010). In terms of delay discounting, it is known that East Asians are less likely than North Americans to discount future rewards (Du et al., 2002; Takahashi et al., 2009; Kim et al., 2012; Ishii et al., 2017). Wang et al. (2016) conducted a large-scale international survey of time preference and suggested the influences of cultural differences in uncertainty avoidance and long-term orientation (Hofstede et al., 2010) on intertemporal choices.

As in the case of delay discounting, cultural differences in social discounting have been shown. Strombach et al. (2014) tested Germans and Chinese for social discounting. The hyperbolic discount function was fitted to the outcomes another person received regardless of cultures. Moreover, although the degree to which the participants discounted another person’s reward did not vary between the cultures, the decrease in the subjective value of another person’s reward was steeper among Germans than among Chinese. Thus, compared to Germans, Chinese were less generous to their closer friends but more generous to distant others. Such a weak effect of social distance in East Asians has also been reported by Buchan et al. (2006), who examined the extent to which participants have other-regarding motives (i.e., trusting others) in an investment game. Whereas North Americans trusted in-group members more than outgroup members, the effect of group membership was weak among Chinese, Japanese, and Koreans. Ito et al. (2011) examined social discounting rates among Japanese and North Americans and found that the discount rate was higher among Japanese than among North Americans not only when the receiver was a relative but also when the receiver was a stranger. In Ito et al. (2011), the hyperbolic discount function was also fitted to another person’s reward in both cultures. Furthermore, a recent study by Ma et al. (2015) showed that in addition to the utility of the hyperbolic discounting model, Chinese who were raised in rural areas were more generous to another person than Chinese who were raised in urban areas. This finding suggests that individuals’ orientation toward others, which is fostered by socioecological environments and not the cultural category, influences an individual’s interpersonal choices.

The previous findings on social discounting are inconsistent with the expected cultural differences based on the well-known cultural dimension of independence and interdependence (or individualism vs. collectivism; Triandis, 1989; Markus and Kitayama, 1991). It is assumed that the self has been characterized as relatively independent and separate from other people in Western cultural contexts and as more interdependent and connected with others in East Asian cultural contexts, and that the cultural dimension fosters psychological processes that vary across different cultures. Based on the cultural dimension, reflecting the interdependence emphasized in East Asian cultures, East Asians are expected to be more generous than are Westerners even in the context of social discounting. However, the previous findings contradict this expectation. Why does such a contradiction occur?

We suggest that the duality of interdependence proposed by Hashimoto and Yamagishi (2016) might be a possible clue to solve the contradiction. They proposed that interdependence consists of one’s tendency to seek social harmony with others to achieve mutual social relationships by considering and responding to others’ feelings and needs (called *harmony-seeking*) as well as one’s sensitivity to negative perceptions and the feelings of others resulting from constraints based on social relationships, where people depend closely on each other (called *rejection avoidance*). It is crucial not to be rejected and ostracized from others in a collective culture in which members are connected with strong ties. Hashimoto and Yamagishi (2016) found that despite the traditional idea that harmony-seeking is a main feature of interdependence that differs across cultures, interestingly, harmony-seeking was higher in North Americans than in Japanese. In contrast, Japanese perceived higher rejection-avoidance than did North Americans. Given that harmony-seeking is crucial for forming mutually cooperative relationships with others, the significance should be universal. Nevertheless, the finding that North Americans perceived higher harmony-seeking than did Japanese suggests that having other-regarding motives is more useful for Westerners than for East Asians with regard to living in their sociocultural environments. In particular, compared to the collective environment in East Asia where interpersonal relationships are relatively stable and fixed so that the cost paid to form new relationships is relatively high, in the sociocultural environment surrounding Westerners which is characterized as mobile and fosters the formation of new relationships, generosity signaling an individual’s good intentions and trustworthiness would be more effective. Accordingly, as generous behaviors attract another person’s attention and enhance the possibility of being chosen as a partner, the utility of generosity should be higher in Western cultures than in East Asian cultures.

### The Present Study

This research sought to examine cultural similarities and differences in social discounting and the role of harmony-seeking in the differences. In Study 1, to find initial evidence for large social discounting among Japanese, we tested Japanese utilizing the same procedure used by Rachlin and Jones (2008, Experiment 3). In Study 2, testing Japanese and Germans and estimating the parameters computed by four social discounting models corresponding to Equations (1)–(4), that is, the exponential, hyperbolic, hyperbolic with exponent, and *q*-exponential models, we examined cultural differences in social discounting and orientation toward harmony-seeking. This examination is novel in terms of two issues. First, we examined the social discounting of not only future gains but also future losses. In delay discounting, the tendency of people to discount future gains more than future losses is called the sign effect (e.g., Frederick et al., 2002). To our knowledge, no study has examined cultural differences in the social discounting of loss and the sign effect. Second, although previous research has suggested the utility of the hyperbolic discounting model across cultures, no study has shown the utility by comparing the hyperbolic discounting model with related models, such as the hyperbolic with exponent model and the *q*-exponential model. Although Takahashi (2013) compared the exponential, hyperbolic, and *q*-exponential models based on social discounting behaviors collected from Japanese, it is unclear whether the findings could apply to social discounting behaviors in another culture. Further, it is unclear whether the utility of the *q*-exponential model could be confirmed by comparison to the hyperbolic with exponent model. Taken together, regardless of cultures, social loss should be discounted less than social gain as the social distance increases. In addition, the subjective value of another person’s outcome should decline inconsistently across people differing in social distance. The decrease should be steeper in the choice for close friends, whereas the decrease should become more moderate in the choice for distant others. In addition to these cultural similarities, we expected that cultural differences in other-regarding motives expressed by harmony-seeking would manifest as a person’s generosity to others in social discounting.

## Study 1

### Materials and Methods

#### Ethics Statement

The study was reviewed and approved by the Experimental Research Ethics Committee at the Graduate School of Humanities, Kobe University. The participants provided a written informed consent at the beginning of the study. All responses were confidential.

#### Participants and Procedure

Ninety-two Japanese undergraduate students (55 females and 37 males, *M*_age_ = 18.78 years, *SD* = 0.78) at a Japanese University participated in this study. They were recruited through a psychology subject pool in the university.

First, following the procedure used in Jones and Rachlin (2006) and Rachlin and Jones (2008), participants were asked to imagine that they created a list of 100 people who were closest to them in the world and placed the people in social distance so that their dearest friend was ranked 1 whereas a mere acquaintance was ranked 100. The participants were then asked to make a series of hypothetical binary choices under the assumption that their choices involved real money. Each choice consisted of two alternatives: (a) The participants themselves would receive a fixed amount of 7,500 yen (about US$75) after a certain period of delay, or (b) a partner at some specified social distance from the participant on the list would receive the fixed amount of 7,500 yen immediately. Option (a) for the participant’s delayed receipt was always presented in the left column, and option (b) for the partner’s immediate receipt was always presented in the right column. The participants were asked to choose whether they preferred option (a) or (b). In option (a), there were 11 delay periods: immediately, 2 days, 5 days, 10 days, 1 month, 2 months, 6 months, 1 year, 2 years, 5 years, and 10 years. For each partner, the 11 delay options for the participant’s receipt of 7,500 yen were compared with the immediate option for the partner’s receipt of 7,500 yen. It was expected that when the participants perceived the value of their delayed receipt of 7,500 yen as small, at some point, they would switch their choices from their delayed receipt to their partner’s immediate receipt. For each partner, the point at which the participant was indifferent between his or her delayed receipt and the partner’s immediate receipt was obtained by averaging the delay of his or her receipt just before he or she switched the choice to the partner’s immediate receipt and the delay of his or her receipt compared with the partner’s immediate receipt immediately after his or her switching of the choice. For example, if a participant preferred the receipt of 7,500 yen with a 5-day delay to the partner’s immediate receipt of 7,500 yen, whereas the participant’s preferred the partner’s immediate receipt of 7,500 yen to a 10-day delay of the participant’s receipt of 7,500 yen, the indifference point was a delay of 7.5 days. The partners varied based on six types of social distance: 1 (i.e., a dearest friend), 2, 10, 20, 50, and 100 (i.e., a mere acquaintance). Thus, six indifference points indicating the length of the delay were computed for each participant. The order of the 11 delay options (ascending or descending) for each partner and the order of the six types of social distance (ascending vs. descending) were counterbalanced across the participants. Accordingly, there were 66 choices in total.

Rachlin and Jones (2008, Experiment 3) proposed an equation regarding the positive association between social distance and the delay of the participant’s reward:

(5) $$D=cN^{1/S},$$

where *D* is the length of the delay, *N* is the social distance, and *c* is substituted for (k_social_/k_delay_)^1/s^. *k* is a discount rate under the assumption of hyperbolic delay discounting and social discounting below:

$$\text{Delaydiscounting}:\text{V}(\text{D})=\frac{\text{V}(0)}{1+{\text{k}}_{\text{delay}}{\text{D}}^{\text{S}}}$$

$$\text{Socialdiscounting}:\text{V}(\text{N})=\frac{\text{V}(0)}{1+{\text{k}}_{\text{social}}\text{N}}$$

where *V*(*D*) is the subjective value of a reward at delay *D*, and *s* is a power-function parameter and suggests individual differences in the sensitivity of *V*(*D*)/*V*(0) to *D*.

In Rachlin and Jones (2008, Experiment 3), *c* was 1.6, and the exponent of Equation (5) (i.e., 1/*s*) was 1.5. These values suggest that the discount rate of social discounting is greater than that of delay discounting, and that the equivalent length of the delay accelerates as the social distance increases.

### Results and Discussion

Following Rachlin and Jones (2008, Experiment 3), the median length of delay across all the participants was computed for each varied partner in the six types of social distance.

By performing a nonlinear regression with *R*, we fitted a model corresponding to Equation (5) to the median length of delay. Accordingly, *c* was 7.09, and the exponent of Equation (5) (i.e., 1/*s*) was 1.35. Thus, compared to the values reported by Rachlin and Jones (2008, Experiment 3), *c* was larger whereas the exponent of Equation (5) was smaller. This larger *c* value suggests that in spite of the common tendency for people to more steeply discount their generosity to their partner compared to their generosity to “their future selves,” Japanese showed a more prominent tendency to do this, compared to the American participants in Rachlin and Jones (2008, Experiment 3). However, the smaller value of the exponent of Equation (5) suggests that the length of the delay accelerated less as a function of social distance in Japanese than in Americans in Rachlin and Jones (2008, Experiment 3).

## Study 2

### Materials and Methods

#### Ethics Statement

The study was reviewed and approved by the Experimental Research Ethics Committee at the Graduate School of Humanities, Kobe University. The participants provided written informed consent at the beginning of the study. All responses were confidential.

#### Participants and Procedure

One hundred twenty-seven German undergraduate students at a German University and 121 Japanese undergraduate students at a Japanese University participated in this study. Because 26 German participants and 2 Japanese participants lacked at least one indifference point due to their misunderstanding of the instruction, their data were excluded from the analyses. Thus, data from 101 Germans (83 females and 18 males, *M*_age_ = 22.71 years, *SD* = 3.16) and 119 Japanese (69 females and 50 males, *M*_age_ = 19.54 years, *SD* = 1.10) were analyzed^1^.

As in Study 1, the participants were initially asked to imagine that they created a list of 100 people who were closest to them in the world and placed the people in various social distances so that their dearest friend was ranked 1 whereas a mere acquaintance was ranked 100 on the list. They were then asked to make a series of hypothetical binary choices on future gains under the assumption that their choices involved real money. Each choice consisted of two alternatives: (a) The participants themselves received a certain amount of money immediately, or (b) a partner at some specified social distance from the participant on the list received the fixed amount of 7,500 yen (or 60 euro) immediately. Option (a) for the participant’s receipt was always presented in the left column, and option (b) for the partner’s receipt was always presented in the right column. The participants were asked to choose whether they preferred option (a) or (b). In option (a), the immediate options varied from 500 to 8,500 yen (or from 4 to 68 euro), in increments of 1,000 yen (or 8 euro). Thus, nine options were prepared for the participant’s receipt. For each partner, the nine options for the participant’s receipt were compared with the immediate option for the partner’s receipt of 7,500 yen (or 60 euro). The point at which the participant was indifferent between his or her receipt and the partner’s receipt was obtained by averaging the amount of the participant’s receipt just before he or she switched the choice to the partner’s receipt and the amount of the participant’s receipt compared with the partner’s receipt immediately after his or her switching of the choice. For example, if a participant preferred to receive 4,500 yen immediately instead of the partner receiving 7,500 yen immediately, whereas the participant preferred the partner receiving 7,500 yen immediately rather than the participant himself or herself receiving 3,500 yen immediately, the indifference point was 4,000 yen. The partners varied based on seven types of social distance: 1 (i.e., a dearest friend), 2, 5, 10, 20, 50, and 100 (i.e., a mere acquaintance). Thus, seven indifference points indicating the amount of receipt were computed for each participant. The order of the nine options for the participant’s receipt (ascending or descending) for each partner and the order of the seven types of social distance (ascending vs. descending) were counterbalanced across the participants. After the participants were asked to choose options regarding future gains for all the types of partners, they were also asked to choose options regarding future losses in the same manner, except that each choice consisted of two alternatives: (a) a certain amount of money was taken away from the participant immediately, or (b) the fixed amount of 7,500 yen (or 60 euro) was taken away from a partner at some specified social distance from the participant on the list immediately. The domain of the choice (gain vs. loss) was thus a within-participant factor. Accordingly, there were 126 choices in total. Future gains and losses were expressed in yen for Japanese participants and converted into euros for German participants, with 1 yen equaling 0.008 euro.

Finally, the participants responded on four independence and interdependence scales (Hashimoto and Yamagishi, 2016), which consist of rejection avoidance (e.g., I find myself being concerned about what others think of me), self-expression (e.g., I always express my opinions in a straightforward manner), harmony-seeking (e.g., I try to respect the feelings of others), and distinctiveness of the self (e.g., I want to live my life differently from others). There were eight statements for each scale. The participants indicated how well each of the statements described them on 7-point Likert-type scales (1 = *doesn’t describe me at all*; 7 = *describes me very much*). The four scales (i.e., rejection avoidance, self-expression, harmony-seeking, and distinctiveness of the self) had reasonable reliabilities in Japan (αs = 0.83, 0.86, 0.66, and 0.66) and Germany (αs = 0.78, 0.82, 0.53, and 0.70).

After performing a nonlinear regression with *R* for each culture, we then fitted the exponential, hyperbolic, hyperbolic with exponent, and *q*-exponential models, which correspond to Equations (1)–(4), respectively, to the mean indifference points across all participants for gain and loss, respectively. We further fitted these models to the mean indifference points in each genotype. The Akaike information criterion (AIC) was used to estimate the goodness of fit. We performed model selection based on the AIC. A smaller AIC indicates a better model fit.

In addition, we computed the area under the curve (AUC) for the gain or loss conditions separately to estimate the extent to which the participants discounted gains or losses. For each culture, social distance and indifference points were standardized by dividing them by the maximum values so that they varied between 0 and 1. Instead of fitting a curve, we connected adjacent delay points by straight lines and computed the area under these lines. Each line made a trapezoid; thus, the total area could be computed by summing the sizes of the trapezoids: (*y*_*i*+1_ + *y_i_*) × (*x*_*i*+1_ – *x_i_*)/2, where *x_i_* and *x*_*i*+1_ indicate social distance (*x*_*i*+1_ is a one-unit farther partner compared to *x_i_*) and *y_i_* and *y*_*i*+1_ are the subjective values of a gain or loss corresponding to these partners. A smaller AUC indicated greater social discounting.

### Results and Discussion

**FIGURE 1:**
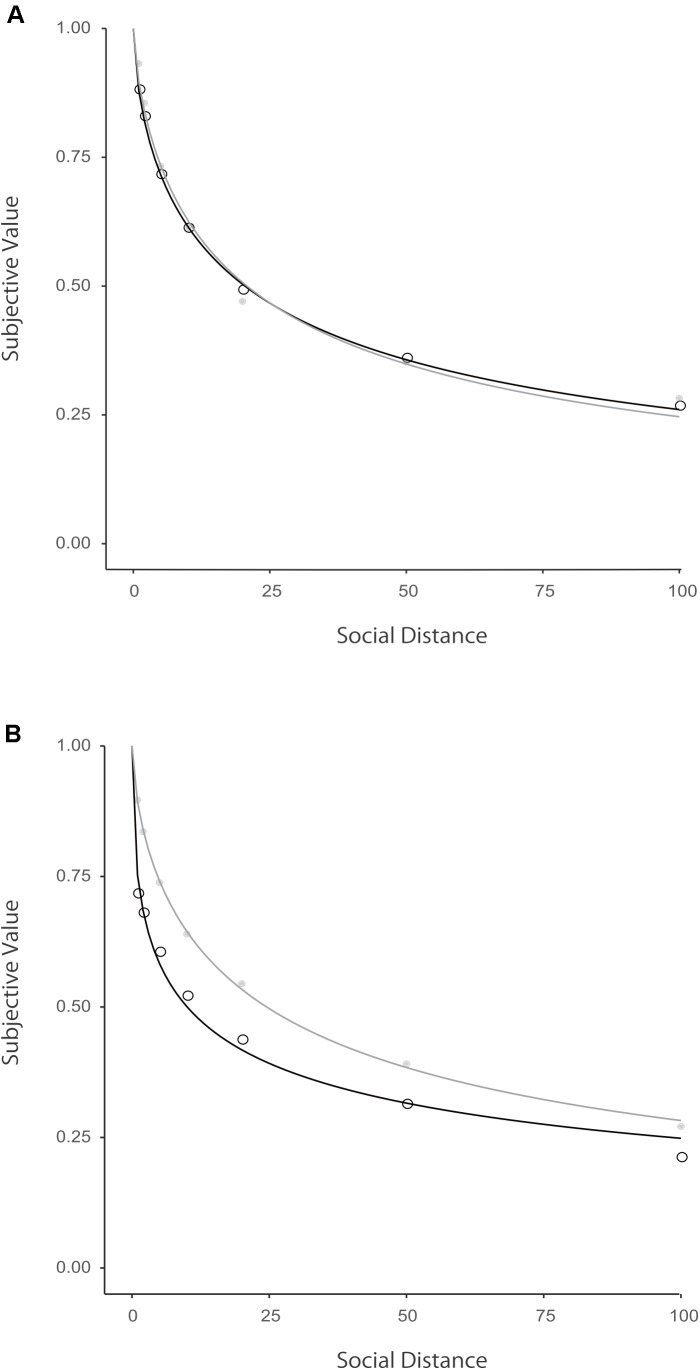
Hyperbolic with exponent functions with social distance for all the participants (German: **A**, Japan: **B**). Mean indifference points were plotted in circle. The curved lines were illustrated in black for gains and in gray for losses.

The AIC and the parameters were estimated for each of the four models. **Table 1** summarizes the results. The AIC values showed that the hyperbolic with exponent model fitted the observed data better than the other three models, except for the loss condition in Germans where the *q*-exponential model fitted the observed data better than the other three models. Overall, Japanese discounted their generosity to their partner more than did Germans in the gain frame, whereas the cultural difference disappeared in the loss frame. In addition, Japanese discounted their generosity to their partner more in the gain frame than in the loss frame, whereas the difference between the two conditions almost disappeared among Germans. **Figure 1** plots the means of subjective value being equivalent to the partner’s fixed amount of gain and loss among Germans and Japanese, which were fitted with the hyperbolic with exponent model. Moreover, the parameter *s* was smaller among Japanese than among Germans for gains and losses. This result suggests that the decrease in a person’s generosity to his or her partner accelerated less as a function of social distance among Japanese than among Germans.

**Table 1:** AIC and parameters for four models.

	Hyperbolic with exponent	Exponential	Hyperbolic	*q*-Exponential
**Gain**				
Germany				
AIC	-45.92	-4.20	-12.89	-34.64
Parameter	*k*_he_ = 0.05	*k*_e_ = 0.03	*k*_h_ = 0.05	*k_q_* = 0.11
	*s* = 0.66			*q* = -1.73
Japan				
AIC	-26.62	0.32	-5.13	-19.00
Parameter	*k*_he_ = 0.10	*k*_e_ = 0.05	*k*_h_ = 0.09	*k_q_* = 0.45
	*s* = 0.48			*q* = -3.01
**Loss**				
Germany				
AIC	-25.95	-4.69	-14.50	-37.58
Parameter	*k*_he_ = 0.05	*k*_e_ = 0.03	*k*_h_ = 0.05	*k_q_* = 0.10
	*s* = 0.71			*q* = -1.42
Japan				
AIC	-43.26	-5.21	-13.42	-28.27
Parameter	*k*_he_ = 0.04	*k*_e_ = 0.02	*k*_h_ = 0.04	*k_q_* = 0.09
	*s* = 0.66			*q* = -1.74

Next, the AUC was submitted to a mixed-model analysis of variance (ANOVA) with one between-subject variable (culture: Germany and Japan) and one within-subject variable (outcome: gain and loss). The results showed a statistically significant main effect of outcome, *F*(1,218) = 5.43, *P* = 0.02, η_p_^2^ = 0.02. The AUC was statistically significantly smaller for gains (*M* = 0.31, *SD* = 0.22) than for losses (*M* = 0.36, *SD* = 0.23). The interaction between culture and outcome was also statistically significant, *F*(1,218) = 6.06, *P* = 0.01, η_p_^2^ = 0.03. **Table 2** presents the relevant means. For gains, the main effect of culture was statistically significant, *F*(1,218) = 5.87, *P* = 0.02, η_p_^2^ = 0.03. The AUC was statistically significantly smaller for Japanese (*M* = 0.29, *SD* = 0.23) than for Germans (*M* = 0.36, *SD* = 0.20). In contrast, for losses, the main effect of culture was not statistically significant, *F*(1,218) = 0.00, *P* = 0.95 (Germans: *M* = 0.36, *SD* = 0.20; Japanese: *M* = 0.36, *SD* = 0.25). In Japanese, the AUC was statistically significantly smaller for gains than for losses, *F*(1,118) = 10.74, *P* = 0.001, η_p_^2^ = 0.08. However, in Germans, there was no difference in the AUC between the two outcomes, *F*(1,100) = 0.01, *P* = 0.92. Moreover, the mean AUCs for gains and losses were highly correlated regardless of culture [Germans: *r*(99) = 0.52, Japanese: *r*(117) = 0.52, *P*s < 0.001].

**Table 2:** Mean AUCs and standard deviations in gain and loss frames in Germans and Japanese.

Frame	Germans		Japanese	
	*M*	*SD*	*M*	*SD*
Gain	0.36	0.20	0.29	0.23
Loss	0.36	0.20	0.36	0.25


As for independence and interdependence (**Table 3**), Germans (*M* = 4.70, *SD* = 1.06) were statistically significantly higher in self-expression than were Japanese (*M* = 4.09, *SD* = 1.20), *t*(218) = 3.96, *P* < 0.001, whereas Japanese (*M* = 5.33, *SD* = 1.09) were statistically significantly higher in rejection avoidance than were Germans (*M* = 3.97, *SD* = 1.13), *t*(218) = 9.07, *P* < 0.001. There was no cultural difference in the distinctiveness of the self (Germans: *M* = 4.50, *SD* = 1.01, Japanese: *M* = 4.59, *SD* = 1.07), *t*(218) = 0.61, *P* = 0.54. Importantly, Germans (*M* = 5.88, *SD* = 0.60) showed higher harmony-seeking than did Japanese (*M* = 5.29, *SD* = 0.79), *t*(218) = 6.11, *P* < 0.001. These patterns were identical to those reported by Hashimoto and Yamagishi (2016) testing North Americans and Japanese. The AUCs for gains and losses were statistically significantly positively correlated with harmony-seeking in Germans [gain: *r*(99) = 0.28, *P* = 0.005. loss: *r*(99) = 0.30, *P* = 0.002] and Japanese [gain: *r*(117) = 0.25, *P* = 0.005. loss: *r*(117) = 0.30, *P* = 0.003). Regardless of culture, those who are higher in harmony-seeking discount their generosity to their partner less in gain and loss frames. However, the AUCs were not statistically significantly correlated with any of the other three scales of independence and interdependence either in the gain frame [for Germans, self-expression: *r*(99) = 0.13, rejection avoidance: *r*(99) = -0.05, distinctiveness of the self: *r*(99) = 0.15; for Japanese, self-expression: *r*(117) = 0.08, rejection avoidance: *r*(117) = -0.08, distinctiveness of the self: *r*(117) = -0.17) or in the loss frame (for Germans, self-expression: *r*(99) = 0.01, rejection avoidance: *r*(99) = -0.03, distinctiveness of the self: *r*(99) = 0.03; for Japanese, self-expression: *r*(117) = 0.14, rejection avoidance: *r*(117) = -0.03, distinctiveness of the self: *r*(117) = -0.14].

**Table 3:** Mean ratings and standard deviations of the aspects of independence and interdependence in Germans and Japanese.

**Aspect**	**Germans**		**Japanese**	
	***M***	***SD***	***M***	***SD***
**Independence**				
Self-expression	4.70	1.06	4.09	1.20
The distinctiveness of the self	4.50	1.01	4.59	1.07
**Interdependence**				
Harmony-seeking	5.88	0.60	5.29	0.79
Rejection avoidance	3.97	1.13	5.33	1.09


We then examined whether harmony-seeking, which varied across cultures, mediates the cultural difference in the AUC for gains. A multiple regression analysis was conducted, in which culture (0 = Japan, 1 = Germany) and harmony-seeking were entered to predict the AUC for gains. Culture is associated with harmony-seeking [*b* = 0.59, *SE* = 0.10, *t*(218) = 6.11, *P* < 0.001] and the AUC for gains [*b* = 0.07, *SE* = 0.03, *t*(218) = 2.42, *P* = 0.02]. When both culture and harmony-seeking were entered simultaneously to predict the AUC for gains, harmony-seeking significantly predicted the AUC for gains [*b* = 0.08, *SE* = 0.02, *t*(217) = 4.00, *P* < 0.001]. On the other hand, the direct path between culture and the AUC for gains was no longer significant, *b* = 0.02, *SE* = 0.03, *t*(217) = 0.79, *P* = 0.43. A bootstrap analysis with a 95% confidence interval (CI; bootstrap sample = 5,000), which was conducted following Preacher and Hayes (2008), revealed a statistically significant indirect effect [CI = (0.02, 0.08)].

## General Discussion

We examined cultural similarities and differences in social discounting and the mediating role of harmony-seeking. Using the same procedure as Rachlin and Jones (2008, Experiment 3), Study 1 showed that Japanese discounted their partner’s outcomes more steeply compared to their own future outcomes. Although the tendency for Japanese was similar to that found for North Americans by Rachlin and Jones (2008, Experiment 3), the former was more obvious than the latter. However, the decrease in the subjective value of the partner’s outcomes, which corresponds to the length of delay to the participants’ receipt of outcomes, accelerated less as a function of social distance among Japanese than among American participants in Rachlin and Jones’s (2008), study (Experiment 3). These patterns imply that compared to Westerners, Japanese show larger social discounting, but their social discounting behaviors are less influenced by the increase in social distance. To examine the cultural similarities and differences in social discounting in more detail, Study 2 tested Japanese and Germans and found that the hyperbolic with exponent model fitted the participants’ discounting behaviors better than the other models, except for the loss condition in Germans, where the utility of the *q*-exponential model was indicated. Moreover, although the social discounting rate was higher in Japanese than in Germans, the cultural difference was limited to the gain frame. Furthermore, we found that in the gain frame an individual’s generosity to another person increased as a function of harmony-seeking, which differs cross-culturally.

Whereas previous research suggested the usefulness of the *q*-exponential model (Takahashi, 2013), the present research indicated the usefulness of the hyperbolic with exponent model cross-culturally. This result advances our understanding of a generalized mathematical model accounting for social discounting behaviors. To verify the validity of the current finding, the usefulness of the hyperbolic with exponent model should be examined further in the future. That said, we should hasten to add that consistent with the findings by Takahashi (2013), the hyperbolic with exponent model and the *q*-exponential model in this research suggest interpersonal inconsistency in social discounting regardless of cultures and frames, which is an important feature for understanding the nature of an individual’s social choice.

Previous research suggested that East Asians are not more generous than Westerners, which is inconsistent with the shared idea that interdependence is more emphasized by East Asians than by Westerners. The current findings are congruent with those from previous research. To solve this inconsistency, we focused on the duality of interdependence proposed by Hashimoto and Yamagishi (2016), according to which interdependence consists of harmony-seeking and rejection avoidance. They found that the latter is higher but the former is lower in Japanese compared to North Americans. As expected, in testing Germans and Japanese, we found that Germans perceive harmony-seeking higher than do Japanese. This finding suggests that Hashimoto and Yamagishi’s (2016) finding could be generalized to another Western culture (Germany). In addition, one advantage of the present research is that we demonstrated that the cultural difference in harmony-seeking could account for the cultural difference in the social discounting of gain. Examination of such an underlying mechanism will contribute to our understating of an individual’s social choice reflecting her or his premise constructed and acquired through her or his living in a given sociocultural environment.

The decrease in a person’s generosity to others was less obvious in Japanese than in Germans. This pattern was similar to that in Strombach et al.’s (2014) comparison of Chinese and Germans. It is also congruent with the findings of Buchan et al. (2006), who showed that the effect of group membership was weaker in Chinese, Japanese, and Koreans than in North Americans in terms of trustworthiness. This result suggests that although Germans are more generous to others in gains than Japanese overall, the cultural difference in generosity is more pronounced among closer friends than among distant others. Thus, whereas Germans might behave generously to specific others to whom they think they can pay a cost (i.e., forego a reward) to maintain friendships with others, the generosity of Japanese might be moderate but relatively fair to acquaintances.

Whereas Japanese discounted social gain more than social loss, which is consistent with Takahashi’s (2013) finding, no difference in social discounting between gain and loss was found among Germans. The Japanese pattern is consistent with the sign effect in delay discounting whereby people are more likely to behave impulsively in gains than in losses. In contrast, as the German participants in this study might have sufficiently high other-regarding motives, place great weight on others’ outcomes, and likely dismiss their own outcome regardless of the frames, a difference between gain and loss might not appear. As this research provided the first evidence on cultural similarities and differences in the social discounting of losses and the sign effect, future work should examine the validity of the current findings in different cultures.

To understand cultural differences in social discounting, it would be informative to explore what socioecological factors influence costly generosity to others. A person’s generosity to others based on sacrificing his or her reward might be more useful in a mobile environment where interpersonal relationships consist of relatively weak ties and showing one’s goodness and trustworthiness through one’s prosocial and generous behaviors are crucial to form and maintain relationships, compared to in a stable environment, where interpersonal relationships are fixed and taken for granted. Moreover, it will also be important to look at whether and to what extent socioecological factors moderate the asymmetry between the social discounting of gains and losses. Residential mobility (Oishi, 2010) and relational mobility (Schug et al., 2010) might be useful for explaining the cultural differences in social discounting.

The present research has several limitations. First, although it followed the manipulation of social distance used in previous research (Jones and Rachlin, 2006; Rachlin and Jones, 2008) and assumed that the representations of social distance do not vary across cultures, we did not verify whether this assumption was correct. Thus, the present research cannot deny the possibility that the relationship type the participants imagined corresponding to a given social distance might differ across cultures, or that the cultural difference in the imagined relationship type might influence the cultural difference in social discounting. Given that in social discounting, a person’s generosity increases when his or her partners are members of mates and genetic kinships (Hackman et al., 2015), the current finding that Germans are more generous than Japanese to others might be because Germans are more likely than Japanese to imagine members of mates and genetic kinships as close friends. Moreover, even if the imagined relationship type does not differ across cultures, perceived emotional closeness to a person at a given social distance might differ between cultures. Hackman et al. (2015) demonstrated that independently of the effect of the relationship type, emotional closeness increases a person’s generosity. This result suggests a possibility that compared to Japanese, Germans perceived greater emotional closeness to others, particularly to closer friends, and that cultural difference led to the Germans’ greater generosity. Future work should focus on these issues. Second, we used hypothetical gains and losses in this study, although Locey et al. (2011) demonstrated that there is no difference in social discounting between real and hypothetical rewards. Another limitation is that we did not consider how the amount of the gains and losses influences social discounting. Rachlin and Jones (2008, Experiment 2) indicated that people discount another person’s reward more as the amount increases. The possibility that the cultural effect in social discounting might change as a function of the amount of gains could be tested in future work.

Despite these limitations, we believe that the present research showing the usefulness of the hyperbolic with exponent model cross-culturally and the mediating role of harmony-seeking in the cultural differences in social discounting in gains contributes to our understanding of the mechanism of individuals’ generosity against the backdrop of the characteristics of sociocultural environments. We hope that future research addresses the generalizability of our findings in divergent cultural contexts.

## Author Contributions

KI designed the research and wrote the paper. KI and CE performed the research and analyzed the data.

## Conflict of Interest Statement

The authors declare that the research was conducted in the absence of any commercial or financial relationships that could be construed as a potential conflict of interest.

## Footnotes

1 The proportion of female participants was significantly higher in Germans than in Japanese, χ^2^ = 14.98, *P* < 0.001. However, no significant effect of gender was found in the analysis of the AUC (*F*s < 2.44, *P*s > 0.11). Thus, gender was excluded in the following analyses.
